# The European Bioinformatics Institute in 2018: tools, infrastructure and training

**DOI:** 10.1093/nar/gky1124

**Published:** 2018-11-16

**Authors:** Charles E Cook, Rodrigo Lopez, Oana Stroe, Guy Cochrane, Cath Brooksbank, Ewan Birney, Rolf Apweiler

**Affiliations:** European Molecular Biology Laboratory, European Bioinformatics Institute (EMBL-EBI), Wellcome Genome Campus, Hinxton, Cambridge CB10 1SD, UK

## Abstract

The European Bioinformatics Institute (https://www.ebi.ac.uk/) archives, curates and analyses life sciences data produced by researchers throughout the world, and makes these data available for re-use globally (https://www.ebi.ac.uk/). Data volumes continue to grow exponentially: total raw storage capacity now exceeds 160 petabytes, and we manage these increasing data flows while maintaining the quality of our services. This year we have improved the efficiency of our computational infrastructure and doubled the bandwidth of our connection to the worldwide web. We report two new data resources, the Single Cell Expression Atlas (https://www.ebi.ac.uk/gxa/sc/), which is a component of the Expression Atlas; and the PDBe-Knowledgebase (https://www.ebi.ac.uk/pdbe/pdbe-kb), which collates functional annotations and predictions for structure data in the Protein Data Bank. Additionally, Europe PMC (http://europepmc.org/) has added preprint abstracts to its search results, supplementing results from peer-reviewed publications. EMBL-EBI maintains over 150 analytical bioinformatics tools that complement our data resources. We make these tools available for users through a web interface as well as programmatically using application programming interfaces, whilst ensuring the latest versions are available for our users. Our training team, with support from all of our staff, continued to provide on-site, off-site and web-based training opportunities for thousands of researchers worldwide this year.

## INTRODUCTION

A primary mission of EMBL-EBI is to collect, organize, add value and make available biomolecular science data to the global life sciences community. To fulfil this mission, we support and provide to our users over 40 data resources (https://www.ebi.ac.uk/services/). These include archival resources storing primary data submitted by researchers and knowledgebases that add value to archival resources through curation and analysis. All data resources are available through web interfaces and most are also accessible using application programming interfaces (APIs) that provide users with high-throughput methods for data access. Additionally, we provide in excess of 150 tools, also through web interfaces and APIs, that allow our users to analyse their own data and data downloaded from EMBL-EBI and other public repositories (1). We describe our tools infrastructure in greater detail below.

A fundamental tenet of our mission is that all hosted data, tools and infrastructure are freely available worldwide, and that data are represented in and shared in a variety of structured and standard formats for consumption by both people and machines. EMBL-EBI’s data resources are part of a worldwide infrastructure of life sciences data resources and many of our resources are run in collaboration with partners around the world, as we described last year (2). Continued international collaboration is crucial to ensuring that the life sciences data infrastructure provides open access archiving and added-value to ever-growing volumes of data submitted to these resources by researchers around the globe. EMBL-EBI is a node in the ELIXIR infrastructure (https://www.elixir-europe.org/) (2) in Europe and is actively engaged in efforts to increase the efficiency of the global infrastructure for life sciences data resources (3).

We continuously review our data services through consultation with our users and monitor new technologies and advances in research in order to ensure that these services provide those users with the resources they need to undertake their work. In this update we summarize growth in our data resources, describe new data services that have been introduced this year and provide an overview of the extensive and broad collection of data analysis tools that complement our data resources.

Our discovery tools and ontologies transcend scientific boundaries, bringing together biologists, clinicians, physicists, mathematicians and software engineers from academia, industry and healthcare. By connecting diverse skills and expertise we are enabling the creative exploration of complex questions in biology today.

## EMBL-EBI DATA RESOURCES IN 2018

**Figure 1.**
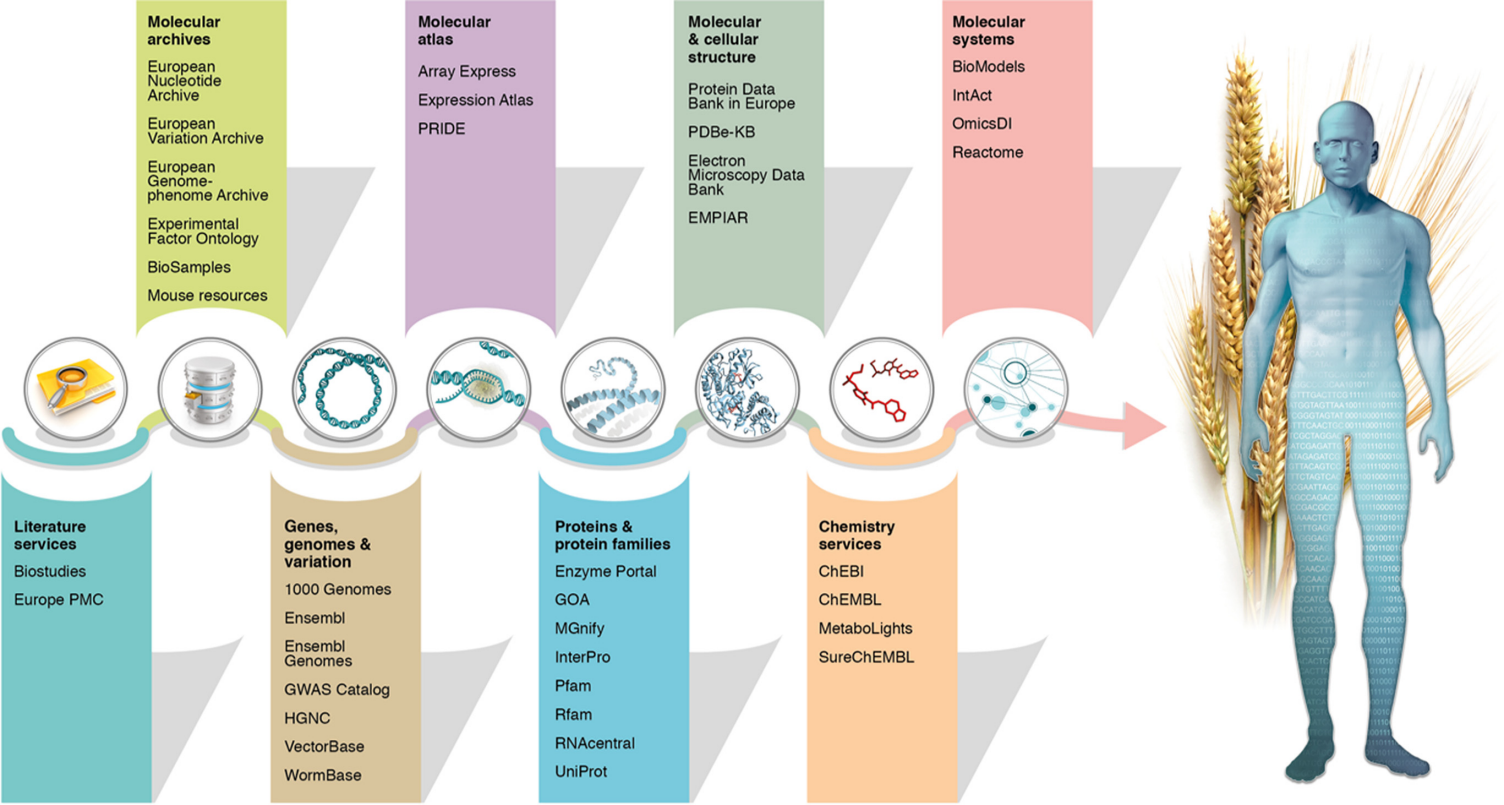
EMBL-EBI data resources. To the left are deposition resources that store primary data submitted by research scientists as well as ontologies and literature resources that span the entire research effort. To the right of the arrow are added-value knowledgebases ordered according to biological scale, from genes to proteins, structures, chemistry and systems. Updates in this NAR Database issue include ArrayExpress (26), BioSamples (27), ChEMBL (28), Complex Portal (29), ENA (30), Ensembl (9), GenCode (31), GWAS Catalogue (32), HGNC (33), InterPro (8), Open Targets (34), PDXfinder (35), Pfam (7), PRIDE (36), RNACentral (37), SIFTS (6) and UniProt (38). Not all EMBL-EBI resources are shown on the figure. For a complete list see https://www.ebi.ac.uk/services.

EMBL-EBI data resources cover the entire range of biological sciences from raw DNA sequences to curated proteins, chemicals, structures, systems, pathways, ontologies and literature. These resources are shown graphically in Figure 1, with the complete list of services available online at https://www.ebi.ac.uk/services. Updates for 18 of these resources also appear in this annual database issue of *Nucleic Acids Research*.

## DATA GROWTH

**Figure 2.**
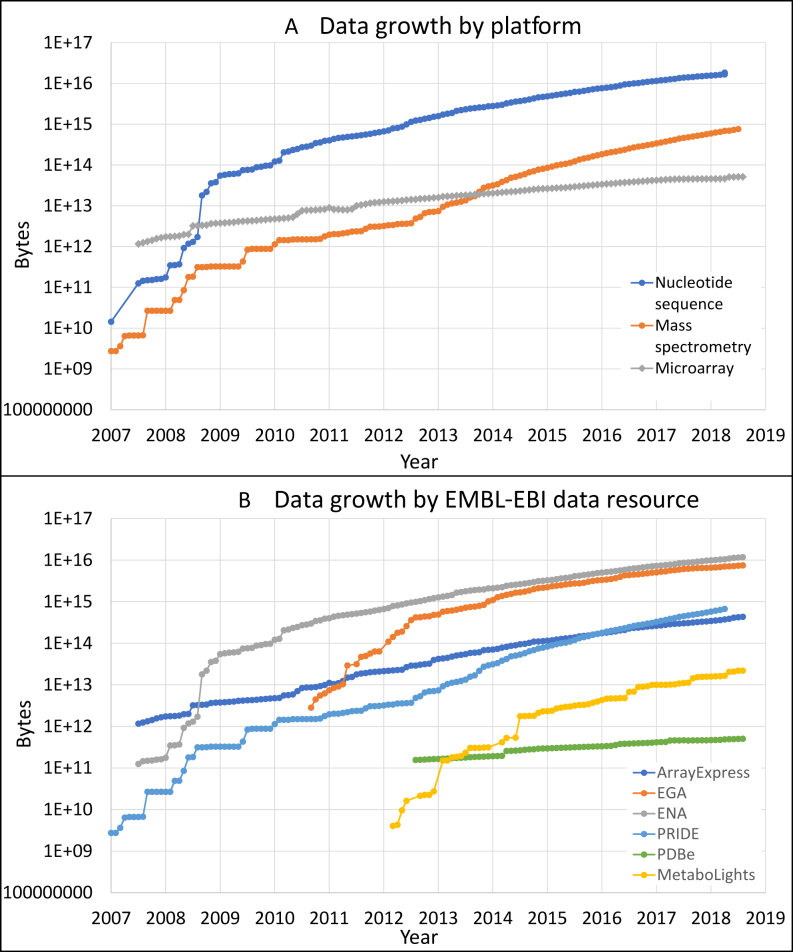
(**A**) Data accumulation at EMBL-EBI by data type: nucleotide sequences, mass spectroscopy and microarray. (**B**) Data accumulation by archive: PRoteomics IDEntifications (PRIDE) (36), European Genome-Phenome Archive (EGA) (39), ArrayExpress (AE) (26), European Nucleotide Archive (ENA) (30), Protein Data Bank in Europe (PDBe) (40) and MetaboLights (16). The *y*-axis for both charts is logarithmic, so most data types are not just growing, but are growing at in increasing rate. In all data resources shown here growth rates are predicted to continue increasing, with notable sustained exponential growth in PRIDE and MetaboLights.

Submissions to our data resources, fuelled by decreasing costs and new technologies, continue to increase exponentially (Figure 2). As always, data volumes are largest for nucleic acid sequences in the European Nucleotide Archive (ENA, https://www.ebi.ac.uk/ena) and the European Genome-phenome Archive (https://ega-archive.org/). We have during the past two years seen notable growth in submissions of mass spectrometry proteomics data to the PRIDE database (https://www.ebi.ac.uk/pride/archive/), and as a category proteomics data has since 2016 had the second largest storage footprint, after nucleotide sequences, among our data resources.

**Figure 3.**
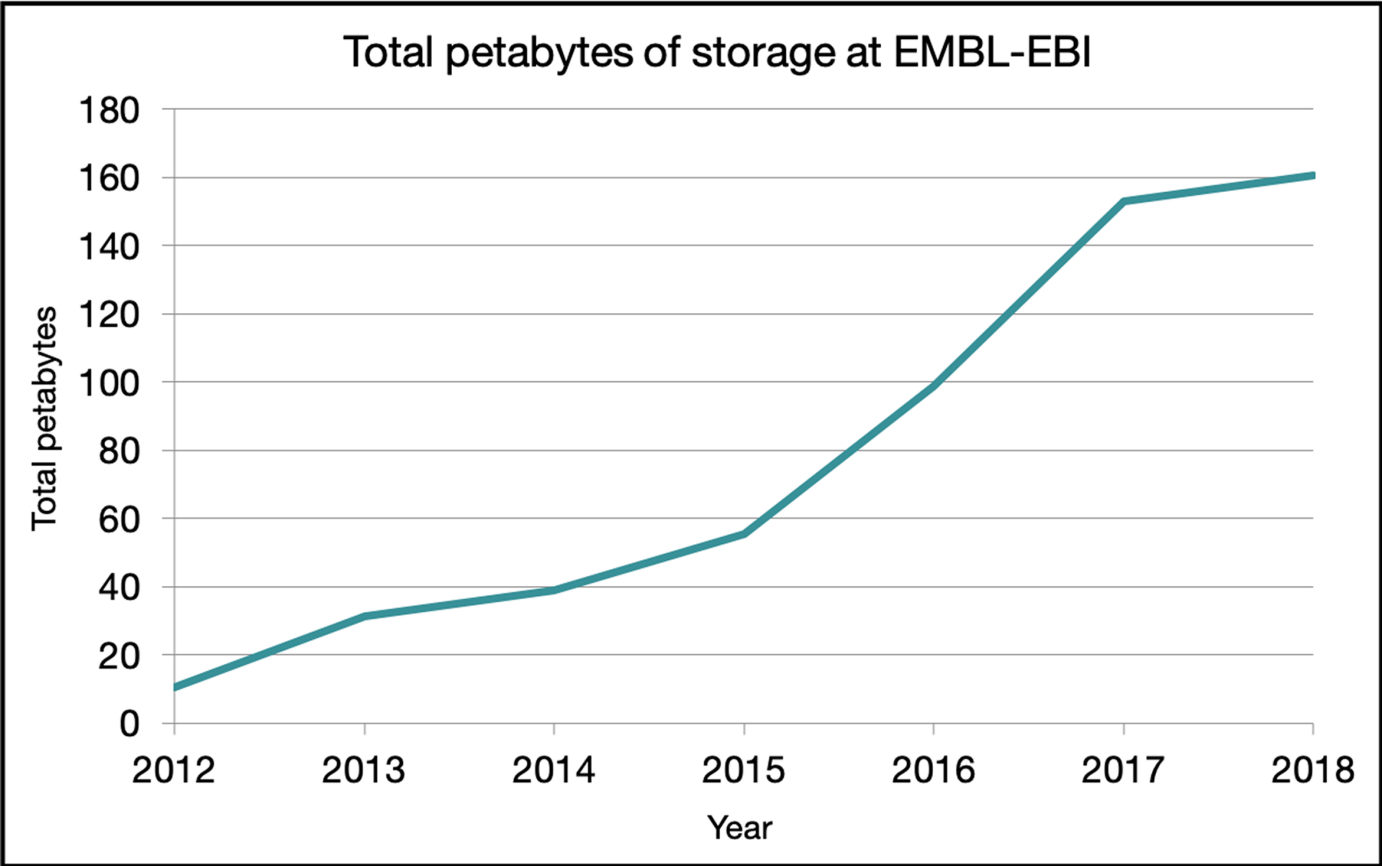
Installed raw storage at EMBL-EBI. The chart shows total installed data storage at EMBL-EBI, including multiple backups for all data resources as well as unused space to handle submissions in the immediate future. The total volume of a single copy of all data resources is roughly 20–25% of the installed storage capacity. Data points (not shown) are the end of each calendar year, thus the range of the *x*-axis is 31 December 2012 through 31 December 2018. Data for end of 2018 are projected based on planned procurement. In 2017, we procured a high volume of disk space at good value that increased capacity substantially, requiring relatively less procurement in 2018. This approach allowed us to utilize our infrastructure budget efficiently.

Our total disk capacity, including all backups and space reserved for immediate future growth, grew from 120 petabytes at the end of 2016 and will reach just over 160 petabytes by the end of 2018 (Figure 3).

The two key components of EMBL-EBI’s provision of all services, both data resources and tools, are interconnectivity and interoperability. As we reported previously, our data resources are internally linked through a dense network of cross references that allow them to share information on related data or biological entities (4). Our unified search engine indexes connections between data resources and provides navigable links between them (5). We continue to expand this search engine, which has now been integrated into over 20 of our data resources, improving the user experience by allowing users to locate, view and download most information and data related to their search term that we have within our data resources.

## NEW DATA RESOURCES AND FEATURES

### Single cell expression atlas

Single cell genomics research has grown exponentially over the past decade as a direct result of advances in technology that allow manipulation and generation of molecular data from individual cells. Researchers worldwide are producing increasing volumes of single cell RNA sequencing (scRNA-seq) data, and there was a clear need for a public repository for these data. In response, EMBL-EBI launched, as part of the existing Expression Atlas, the Single Cell Expression Atlas (http://www.ebi.ac.uk/gxa/sc) as a new added-value database and web service (https://www.ebi.ac.uk/gxa/home) to collect raw data and metadata from single cell RNA-Seq studies. The Single Cell Atlas’ pipelines annotate and re-analyse data from publicly available studies, store them in standardized formats and allow searching and display of gene expression in individual cells from different tissues or experimental conditions, such as diseases. The Single Cell Expression Atlas currently contains data for nine species: *Anopheles gambiae, Caenorhabditis elegans, Danio rerio, Drosophila melanogaster, Homo sapiens, Mus musculus, Plasmodium berghei, Rattus norvegicus* and *Schistosoma mansoni*. Datasets from additional species, as well as more functionality for their exploration on the web, will be made available over new releases in due course. The resource will also host datasets from the Human Cell Atlas (https://www.humancellatlas.org/) as these become available (https://www.humancellatlas.org/files/HCA_WhitePaper_18Oct2017.pdf).

### Protein Data Bank in Europe—knowledge base

Protein Data Bank in Europe—Knowledge Base (PDBe-KB; pdbe-kb.org) is a community-driven resource managed by the PDBe team (http://www.ebi.ac.uk/pdbe/), collating functional annotations and predictions to provide biological context for structure data in the PDB archive. PDBe-KB is a collaborative effort between PDBe and a diverse group of bioinformatics resources and research teams. PDBe-KB consolidates into one resource the curated and enriched data from a number of data projects, briefly described below.

Structure Integration with Function, Taxonomy and Sequence (SIFTS, http://www.ebi.ac.uk/pdbe/docs/sifts/) provides residue-level mappings between UniProtKB (https://www.uniprot.org) and PDB entries, allowing for easy transfer of value-added annotations between protein structures and sequences (6). In that vein, SIFTS provides cross-references to the IntEnz (https://www.ebi.ac.uk/intenz/), GO (http://geneontology.org), Pfam (7), InterPro (8), SCOP (http://scop.mrc-lmb.cam.ac.uk/scop/), CATH (http://www.cathdb.info), PubMed (https://www.ncbi.nlm.nih.gov/pubmed/), Ensembl (9) and Homologene (https://www.ncbi.nlm.nih.gov/homologene) resources. The information is updated and released every week at the same time as the release of new PDB entries and is widely used by resources such as RCSB PDB (https://www.rcsb.org), PDBsum (www.ebi.ac.uk/pdbsum), Pfam, CATH, SCOP2 (http://scop2.mrc-lmb.cam.ac.uk), InterPro, MobiDB (http://mobidb.bio.unipd.it) and others. Recently, SIFTS was revised to include mappings to UniRef90 (https://www.uniprot.org/help/uniref) clusters, expanding the applicability of structures from 44 000 to nearly two million UniProtKB records.

The FunPDBe project (https://www.ebi.ac.uk/pdbe/funpdbe/deposition/) integrates and makes available structural and functional annotations for macromolecular structure data in the Protein Data Bank (PDB, https://www.wwpdb.org). It is a collaboration between the Protein Data Bank in Europe (PDBe) and world-leading providers of structural bioinformatics data, who contribute computationally predicted or manually curated information on functional sites, enzyme function and effects of variants and mutations.

A collaboration with Genome3D (http://genome3d.eu) and InterPro teams will lead to inclusion of evolutionary protein residue conservation information into PDBe-KB.

While many RNA molecules are classified by Rfam, a database of non-coding RNA families based at the EMBL-EBI (http://rfam.xfam.org), this information was not accessible to the users of PDBe. Over the past year the PDBe and Rfam teams collaborated to make use of the mappings of RNA molecules present in the PDB, enabling more accurate search of relevant PDB data. As part of PDBe-KB, the project will continue to work on other features, such as improved representation and visualization of RNA molecules.

PDBe-KB is developing adequate infrastructure to support the deposition of value-added annotations from partners worldwide and programmatic tools to disseminate the assembled information. For example, much of the SIFTS information is already available via the PDBe API.

Information encoded in the PDB structures is very complex and requires tools to visualize it in a manner accessible to experts and non-experts alike. Thus, intuitive visualization of structures and associated annotations is a focus area for PDBe-KB. In collaboration with the UniProt and InterPro teams, PDBe-KB is improving the ProtVista tool for the visualization of protein sequences (10), which will be shared across EMBL-EBI. ProtVista will interact with the 2D protein topology viewer and with the LiteMol viewer (http://webchemdev.ncbr.muni.cz/Litemol/) for 3D structure and supporting experimental data. The latter two viewers are encapsulated as re-usable web-components that can be easily integrated in any website, and ProtVista will be likewise encapsulated. Similarly, PDBe-KB is developing a web-component to interactively display simplified 2D representations of small molecules in the PDB, and in particular of their binding environments and interactions within macromolecular assemblies. This new component will also interact with the 3D LiteMol viewer, allowing the user to simultaneously view the 2D schematic representation and the full 3D environment. A further web-component under development will display the information on quality of PDB structures with an interactive Ramachandran plot, synchronized with other web-components mentioned above.

### Europe PMC annotations platforms and preprints

Europe PMC (11), a comprehensive resource of biomedical research publications, has added preprint abstracts to its search results, augmenting the results already returned for peer-reviewed content. Preprints are articles that have not been peer-reviewed, edited, typeset or published in a journal. They enable researchers to share their scientific findings freely and quickly with the scholarly community and to receive feedback before submitting a paper to a journal. Nearly 48 000 preprints from bioRxiv, PeerJ Preprints, ChemRxiv, Preprints.org, F1000Res and the Open Research platforms powered by F1000 (Gates Open Res, Wellcome Open Res, HRB Open Res, AAS Open Res and MNI Open Res) can be discovered in Europe PMC. Preprints in Europe PMC are enriched in a number of ways: by providing links to final peer reviewed publications, including them in Europe PMC routine text mining processes, and providing tools for claiming preprints to the author’s ORCID.

Along with expanding content coverage to include preprints, Europe PMC has established a community annotation platform to facilitate knowledge extraction from life sciences literature. The platform consolidates text-mined outputs from various providers and makes them available both via the Europe PMC website as text highlights using the SciLite application (12), and programmatically, with the Europe PMC Annotations API. Over 500 million annotations extracted from biomedical abstracts and open access full text articles are available in Europe PMC. The text-mined concepts cover a variety of biological entities, events and relations: gene names, organisms, gene-disease associations, protein interactions as well as data citations in the form of accession numbers and data DOIs. The annotations platform enables researchers to discover evidence, locate primary data and harness the power of text mining for the benefit of their own research.

### BioStudies new ingest sources and features

We continued further development of BioStudies, which organizes data associated to a publication (13). A data filtering and faceting mechanism was added, facilitating exploration within a large set of studies; facets can be defined on a per-project basis. Scalability improvements to all components enable deposition and access to very large studies comprising thousands of data files. Data submitters get a link to their private studies, enabling peer review on embargoed datasets prior to publication.

### New PRIDE Archive and increase in proteomics data re-use

PRIDE Archive (https://www.ebi.ac.uk/pride/archive/, the archival component of PRIDE) and the related data submission framework have been further developed to support the increase in submitted data volumes (about 275 submitted datasets per month in 2018, see Figure 2) and additional use cases. A new scalable and fault tolerant storage backend, API, and web interface have already been implemented as a part of an ongoing process to redevelop the whole PRIDE infrastructure. Notably, improved support for the deposition of quantitative proteomics data isna now available via the increasingly popular mzTab format (14). As a result of an unprecedented amount of proteomics data in the public domain, data re-use of proteomics data continues to grow steadily (15). In this context, PRIDE is increasingly disseminating high-quality proteomics data to the following EMBL-EBI resources: (i) Ensembl, where a ‘TrackHub’ based mechanism enables integration of peptide sequences into the genome browser. For downstream analysis, all data (mainly human at present) can be downloaded from http://ftp.pride.ebi.ac.uk/pride/data/proteogenomics/latest/archive/; (ii) Expression Atlas, where around 15 quantitative proteomics datasets, re-analysed using a MaxQuant-based pipeline (16), have already been integrated with the existing transcriptomics datasets; and (iii) UniProt, where the focus is increasingly being put in supporting experimental observations post-translational modifications.

## TOOLS

In addition to data resources EMBL-EBI also provides over 150 bioinformatics tools to our users (https://www.ebi.ac.uk/services) (1). These are mainly sequence analysis tools that are fundamental to the research community and complement public data repositories and comprise the workhorses of bioinformatics. We define a tool as a web-based user interface that carries out an analysis or computation on data supplied by a user. Often, users are analysing data downloaded from EMBL-EBI resources, hence many of our data resources provide direct links to commonly used tools. All these tools are accessible from our services page (https://www.ebi.ac.uk/services). Most of the tools are also available to download for installation onto local systems, either directly or using containers.

Many tools, and most data resources, are accessible programmatically through APIs. EMBL-EBI provides APIs for most data services. Most of these APIs are compliant with the OpenAPI specification (https://www.openapis.org) and and are designed for easy integration into analysis pipelines using the CWL (Common Workflow Language) specification (https://doi.org/10.7490/f1000research.1110021.1). A current list of these APIs is available from https://www.ebi.ac.uk/seqdb/confluence/display/JDSAT/Data+Retrieval+Web+Services.

Our goal in providing tools is to allow our users to extract as much value and information as possible from data they generate in their laboratories, download from our data resources, or download from our collaborators. In pursuit of that goal we work to improve our users’ experience by ensuring that these tools are always up-to-date by optimizing their usability both through web interfaces and automated pipelines, and by continually investing in maintaining and upgrading our infrastructure to ensure that computational analyses are accurate and results are delivered to users quickly.

Most of our tools were developed by EMBL-EBI and by our collaborators, for example InterProScan (17) and Clustal Omega (18), whereas others have been developed elsewhere, then adapted for use within our infrastructure, such as HMMER (19) and BLAST+ (20) Regardless of their provenance, we actively manage all tools to ensure that coverage, in terms of the analytical capability they represent, is in line with current user needs and bioinformatics practises.

Some tools are also linked directly from search results, allowing users to analyse results with one click. Thus, a protein search in UniProt provides a link that sends one or more proteins selected from within the UniProt search result directly to BLAST+ for comparison to other known proteins, and separate link sends selected proteins from the search result for alignment with Clustal Omega. These are good examples of the integration of the JDispatcher APIs into third party web portals.

EMBL-EBI data resources are also used by our own data resources. For example, many resources use sequence similarity tools, notably BLAST+ and HMMER, to classify sequences, and all protein sequences in UniProt are processed using InterProScan7 to classify to which protein family they belong.

EMBL-EBI tools are extensively documented, with documentation available on the home page of each tool that explains how to use the web interface and, for most tools, the API. Documentation is now enriched with examples of the types of data and formats required to run each tool. The EMBL-EBI training programme (see section below) also provides instruction and materials in using tools for in-person and online resource-specific courses.

The set of tools provided by EMBL-EBI also includes various means of retrieving data from our data resources to match the needs of our users. Conceptually, there are two types of data retrieval: *mapping* and *retrieval.* Mapping services, such as https://www.identifiers.org, allow users to determine whether our resources have data of interest to them, and where those data are. Retrieval services are those that allow users to actually retrieve data for use in their research. The quintessential example of a retrieval service is the Dbfetch tool (https://www.ebi.ac.uk/Tools/dbfetch), which was the first tool to run from EMBL-EBI’s website in 1996, and which is still used millions of times per year.

Usage of sequence analysis tools has been steadily growing. As of mid 2018 EMBL-EBI runs an average of 250 000 jobs—where a ‘job’ is an analysis call to one of our tools—per day, with spikes of up to 500 000 in 1 day occurring often. The 10 most highly used tools are InterProScan; the BLAST+ suite; HMMER’s phmmer and hmmscan; Clustal Omega; *needle* and *water* from EMBOSS (21) for pairwise alignments, and translation tools such as transeq and six pack, also from EMBOSS. For sequence similarity searches, the most often used sequence libraries are UniProtKB; Pfam; UniProtKB/Swiss-Prot; PDB, Ensembl Genomes (22); UniProt reference proteomes; IMGT/HLA (23) and all the ENA nucleotide sequences classes.

## INFRASTRUCTURE

As we noted above, EMBL-EBI’s computational infrastructure is continually expanding in order to manage increasing data submissions. In addition to this expansion we also manage our infrastructure to improve services. During the past year have increased our compute capacity to run our analytical tools whilst dealing with an ever-increasing number of users. In the first quarter of 2018 the main compute farm had 34 000 cores (27 000 high throughput and 7000 high performance), and our internal network currently has a 100 Gigabit backbone within its data centres, with multiple 10 Gigabit connections between data centres.

These infrastructure improvements resulted in up to 50% faster completion of computationally intensive analytical jobs, such as BLAST+ searches. We also increased EMBL-EBI’s connection to the internet backbone from a single 10 Gigabit link to dual 10 Gigabit links. This significantly improved response times for all of our users, including returning completed analytical jobs to them.

Complementing these improvements in our physical infrastructure is the expanded coverage of our unified search engine (5), which has now been integrated into over 20 portals at EMBL-EBI. The net effect of this is improvements in the user experience by allowing users to locate, view and download all information and data related to their search term.

## TRAINING

Training is at the heart of EMBL-EBI’s mission and an important means of supporting our users. As noted in the section on data growth above, the volume and variety of data generated by molecular life scientists continues to increase; single cell sequencing, imaging over multiple scales and the application of biomedical informatics in clinical practice are just some of the advances that fuel the ongoing need for training. The mission of EMBL-EBI’s training programme is to enable EMBL to deliver world-leading training in bioinformatics and scientific service provision to the research community, empowering scientists at all career stages to make the most of biological data and strengthening bioinformatics capacity across the globe.

EMBL-EBI’s training programme (www.ebi.ac.uk/training/) has five major strands:

- *Training on-site*: delivering courses to develop practical skills and knowledge, led by EMBL-EBI experts and hosted in our purpose-built training suite.
- *Training off-site*: our trainers travel to host organizations worldwide to provide hands-on training on EMBL-EBI data, tools and resources.
- *Visits and secondments*: visiting scientists are embedded in an EMBL-EBI group for up to 6 months, often leading to longer-term collaboration.
- *Training online*: we provide free access to EMBL-EBI courses, allowing individuals to choose when, where and how they learn; this is complemented by regular live webinars.
- *Trainer support*: we help to strengthen bioinformatics training capacity, working with communities of bioinformaticians to develop relevant and high-quality training courses, supported by EMBL-EBI experts and materials.

This combination of activities allows us to deliver both quality and scalability: in 2017 we participated in 341 training and outreach events that supported biomedical and life-science professionals throughout the world. Train online was accessed by 400 240 unique IP addresses. We expanded our online training offerings, adding 11 new courses and 38 webinars, and we delivered six train-the-trainer events, training 61 bioinformatics instructors.

In October 2017 we launched CABANA (www.cabana.online)—an exciting new project funded by the Global Challenges Research Fund (https://www.ukri.org/research/global-challenges-research-fund/), that aims to address the slow implementation of data-driven biology in Latin America by creating a sustainable capacity-building programme. With an international consortium of 10 organizations—nine in Latin America, the EMBL-EBI-led CABANA project is combining research secondments, workshops, train-the-trainer activities and new eLearning content to strengthen research in three challenge areas of particular relevance to Latin America—communicable disease, sustainable food production and protection of biodiversity.

Evaluating the quality, reach and impact of training is a significant challenge; we have put considerable effort into developing meaningful impact measures for our face-to-face training, and our approach to impact assessment is maturing. We consider our training to have impact when:

- Trainees are able to undertake appropriate analysis/use resources and tools introduced during the course.
- The ability to use what they have learned has enabled or improved their ability to do research.
- They have transferred the knowledge and skills gained to others.
- The training programme actively contributes to EMBL’s science.

## LOOKING AHEAD

We are continually assessing the needs of life sciences researchers worldwide and work to create new sustainable open access resources when new data types are mature. For example, we described last year (2) the need for an archive to store reference images for all imaging scales, and this resource, the BioImage Archive is now being established (24).

In addition to creating new resources we will continue to grow our existing data resources, and to support their users, by ensuring that they continue to accept, curate and analyse research-generated data, and that our technical infrastructure (computational capacity, data storage and connection to the worldwide web), grow in step with data depositions and usage of those resources. Supporting data resources is a collaborative effort of many institutions and funders worldwide, and we continue working with our users and other life science data resources to ensure that global life sciences data resources are managed efficiently across to maximize efficient use of resources.

EMBL-EBI’s data resources play an increasingly important role in facilitating the translations of omics technologies from the research domain into medical practice. These activities focus in particular on the provision of reference data for clinical research and the development of data structures and standards that promote understanding of disease mechanisms, accurate diagnoses and more targeted treatments. In the near future we will increase support for clinical researchers and practising healthcare professionals with the provision of bioinformatics services to analyse patient genome data, for example, with further development of tools such as the Ensembl Variant Effect Predictor (25), which provides a toolset for the analysis, annotation and prioritization of genomic variants in coding and non-coding regions. EMBL-EBI also provides technology leadership in workstreams within the Global Alliance for Genomics and Health (https://www.ga4gh.org/), the international coalition formed to enable standards for the responsible sharing of genomics data in healthcare.
